# Blockade of MCAM/CD146 impedes CNS infiltration of T cells over the choroid plexus

**DOI:** 10.1186/s12974-018-1276-4

**Published:** 2018-08-22

**Authors:** Johanna Breuer, Eva Korpos, Melanie-Jane Hannocks, Tilman Schneider-Hohendorf, Jian Song, Lisa Zondler, Sebastian Herich, Ken Flanagan, Thomas Korn, Alexander Zarbock, Tanja Kuhlmann, Lydia Sorokin, Heinz Wiendl, Nicholas Schwab

**Affiliations:** 1 Clinic of Neurology with Institute of Translational Neurology, University of Münster, Albert-Schweitzer-Campus-1, Building A01, 48149 Münster, Germany; 2 Institute of Physiological Chemistry and of Pathobiochemistry, University of Münster, Münster, Germany; 3 Cells-in-Motion Cluster of Excellence, University of Münster, Münster, Germany; 4 Department of Anesthesiology, University of Münster, Münster, Germany; 5 Prothena Biosciences Inc., South San Francisco, CA USA; 6 Department of Neurology, Technical University of Munich, Munich, Germany; 7 Munich Cluster for Systems Neurology (SyNergy), Munich, Germany; 8 Department of Neuropathology, University of Münster, Münster, Germany

**Keywords:** MCAM, VLA-4, CNS-migration, Choroid plexus, Laminin 411, EAE

## Abstract

**Background:**

Very late antigen 4 (VLA-4; integrin α4β1) is critical for transmigration of T helper (T_H_) 1 cells into the central nervous system (CNS) under inflammatory conditions such as multiple sclerosis (MS). We have previously shown that VLA-4 and melanoma cell adhesion molecule (MCAM) are important for trans-endothelial migration of human T_H_17 cells in vitro and here investigate their contribution to pathogenic CNS inflammation.

**Methods:**

Antibody blockade of VLA-4 and MCAM is assessed in murine models of CNS inflammation in conjunction with conditional ablation of α4-integrin expression in T cells. Effects of VLA-4 and MCAM blockade on lymphocyte migration are further investigated in the human system via in vitro T cell transmigration assays.

**Results:**

Compared to the broad effects of VLA-4 blockade on encephalitogenic T cell migration over endothelial barriers, MCAM blockade impeded encephalitogenic T cell migration in murine models of MS that especially depend on CNS migration across the choroid plexus (CP). In transgenic mice lacking T cell α4-integrin expression (CD4::*Itga4*^*−/−*^), MCAM blockade delayed disease onset. Migration of MCAM-expressing T cells through the CP into the CNS was restricted, where laminin 411 (composed of α4, β1, γ1 chains), the proposed major ligand of MCAM, is detected in the endothelial basement membranes of murine CP tissue. This finding was translated to the human system; blockade of MCAM with a therapeutic antibody reduced in vitro transmigration of MCAM-expressing T cells across a human fibroblast-derived extracellular matrix layer and a brain-derived endothelial monolayer, both expressing laminin α4. Laminin α4 was further detected in situ in CP endothelial-basement membranes in MS patients’ brain tissue.

**Conclusions:**

Our findings suggest that MCAM-laminin 411 interactions facilitate trans-endothelial migration of MCAM-expressing T cells into the CNS, which seems to be highly relevant to migration via the CP and to potential future clinical applications in neuroinflammatory disorders.

**Electronic supplementary material:**

The online version of this article (10.1186/s12974-018-1276-4) contains supplementary material, which is available to authorized users.

## Background

Multiple sclerosis (MS), a chronic disorder of the central nervous system (CNS), is characterized by inflammatory lesions at early disease stages that are caused by the infiltration of autoreactive immune cells into the CNS [1, 2]. The inflammatory infiltrates, containing pathogenic T cells (T helper 1 (T_H_1) and T_H_17 cells), lead to demyelination and neuronal degeneration resulting in serious physical disability [3, 4]. Immune cell infiltration from the periphery into the CNS, characteristic of the common relapsing-remitting form of MS (RRMS), has been successfully targeted by new therapies. In particular, the monoclonal antibody natalizumab, which recognizes the integrin α4 subunit of very late antigen-4 (VLA-4, integrin α4β1, CD49d/CD29), has proven highly effective in reducing disease progression by preventing leukocyte migration into the CNS [5, 6]. VLA-4 plays a key role in the entry of encephalitogenic T cells into the CNS by mediating the initial rolling and adhesion steps of transmigration through interaction with its receptor, vascular cell adhesion molecule-1 (VCAM-1), expressed on endothelial cells of post-capillary venules upon inflammation [7]. Even though VLA-4 has been shown to play a crucial role in leukocyte trafficking over the blood-brain barrier (BBB), animal studies revealed that a subpopulation of T_H_17 cells, which represent a major pathogenic T cell population in murine experimental autoimmune encephalomyelitis (EAE) [8–10], can invade the CNS in a VLA-4 independent manner. Recent studies suggest that T_H_17 cells mainly rely on leukocyte function-associated molecule-1 (LFA-1) mediated firm adhesion to endothelial intercellular adhesion molecule-1 (ICAM-1) to penetrate the choroid plexus (CP) and enter into the CNS parenchyma or the cerebrospinal fluid (CSF) space [11–13]. However, LFA-1 is primarily involved in the firm adhesion to endothelium and subsequent para-endothelial migration, and not in the initial rolling and adhesive steps [14]. This poses the question of how the initial adhesive steps are mediated in the encephalitogenic T_H_17 subpopulation and raises the possibility that different immune cell-adhesion receptor interactions are employed, depending on the route of entry into the brain and the cellular barrier involved. Three important barriers restrict access of circulating immune cells to the CNS parenchyma: the endothelial BBB in CNS post-capillary venules, the epithelial-cerebrospinal fluid barrier (BCSFB) at the CP, and the arachnoidea formed by leptomeningeal cells [14–16].

Apart from LFA-1–ICAM-1-dependent penetration of the CP by T_H_17 cells, we have recently shown that adhesion of T_H_17 cells to an in vitro model of the BBB in the absence of VLA-4 involves melanoma cell adhesion molecule (MCAM; CD146) [17]. MCAM is an extracellular adhesion molecule broadly expressed on mesenchymally derived tissues [18]; in humans it has also been reported to be expressed on a subpopulation of IL-17-secreting CD4^+^ and CD8^+^ T cells (T_H_17 and Tc17 cells, respectively) [18–25]. EAE experiments performed in endothelial MCAM knockout mice have shown reduced disease severity and reduced T_H_17 infiltration into the CNS, supporting a role for this molecule in the entry of encephalitogenic T cells into the brain [26]. Laminin 411 (composed of α4, β1, and γ1 chains), a major component of endothelial basement membranes [27], has been identified as a ligand for MCAM, and MCAM-laminin 411 interactions have been proposed to facilitate penetration of the BBB [22]. However, whether MCAM can contribute to T_H_17 penetration of other CNS barriers remains unclear.

The focus of the current study was, therefore, to elucidate the pathogenic relevance of MCAM compared to VLA-4 in CNS inflammation in vivo by using different murine models of MS (active and spontaneous experimental autoimmune encephalitis (EAE)). Using antibody- mediated blocking of VLA-4 and/or MCAM and T cell-specific ablation of α4-integrin expression, we show here that VLA-4 blockade abrogates the encephalitogenic potential of T cells in a wide-ranging way by blocking migration over endothelial barriers, whereas MCAM targeting primarily restricts migration of T_H_17 cells over the CP into the CNS by inhibiting MCAM-laminin α4 interactions at the endothelial CP layer.

## Methods

### Mice

Eight- to 10-week-old C57BL/6 mice were purchased from Harlan Laboratories (Horst, Netherlands). CD4-cre mice were crossed with integrin *Itga4*^flox/flox^ mice as previously described [12, 28] to generate mice lacking integrin α4β1 specifically on T cells (CD4::*Itga4*^−/−^). Double-transgenic mice (TCR^MOG^ × IgH^MOG^ mice or OSE/DEVIC mice) expressing T- and B-cell receptors that recognize the same myelin protein (MOG_35–55_) were generated by cross- breeding transgenic mice carrying either a MOG_35–55_-specific T cell receptor (TCR^MOG^ also referred to as 2D2 [29]) or a MOG_35–55_-specific B-cell receptor (IgH^MOG^ also referred to as Th [30]) as described previously [31, 32].

All animal experiments were approved by and conducted in accordance with the laws and regulations of the regulatory authorities for animal care and scientific use in North Rhine-Westphalia, Germany (TVA-number 84-02.04.2014.A075).

### Induction of EAE

EAE was induced in 6- to 8-week-old CD4::*Itga4*^−/−^ mice by subcutaneous injection of 200 μg MOG_35–55_ peptide (MEVGWYRSPFSRVVHLYRNGK; Charité, Berlin, Germany) emulsified in CFA containing 200 μg *Mycobacterium tuberculosis* H37RA (Difco, MI, USA). Pertussis toxin (400 ng; Alexis, San Diego, CA, USA) in 200 μl PBS was injected intraperitoneally (i.p.) on the day of immunization (day 0) and 2 days later. Disease severity was scored daily on a scale from 0 to 10 as previously described [33]: grade 0, no abnormality; grade 1, limp tail tip; grade 2, limp tail; grade 3, moderate hindlimb weakness; grade 4, complete hindlimb weakness; grade 5, mild paraparesis; grade 6, paraparesis; grade 7, heavy paraparesis; grade 8, tetraparesis; grade 9, quadriplegia or premoribund state; grade 10, death (experimental autoimmune neuritis (EAN) score). Animals were scored in a blinded fashion by two independent observers. Disease onset was defined as a score greater than or equal to 1.

### Antibody treatment for in vivo blocking

The blocking antibodies anti-mMCAM (clone 15) (described in [22]) and α4 integrin neutralizing antibody (clone: PS/2, BioXCell, New Hampshire, USA) as well as the appropriate isotype control antibodies (rat IgG1, clone: HRPN and rat IgG2b, clone: LTF-2; both BioXCell) were used at a concentration of 10 mg/kg body weight. Mice were treated every other day with i.p. injections of the respective antibody from the indicated day on.

### Cell preparation and flow cytometry

Single-cell suspensions of mouse spleens and peripheral blood were prepared as described previously [33]. Mononuclear cells were isolated from spinal cord and brain by Percoll gradient [33] and cells were stained for 30 min at 4 °C with fluorescence-labeled mAbs in PBS containing 0.1% BSA. The following antibodies were used for the detection of cell surface markers: anti-MCAM (clone: ME-9F1), anti-CD3 (clone: 17A2), anti-CD4 (clone: RM4-5), anti-NK1.1 (clone: PK136) (all from BioLegend, Fell, Germany). Cells were assessed on a Gallios™ (Beckman Coulter, Krefeld, Germany) and analyzed using Kaluza software (Beckman Coulter).

### Adoptive cell-transfer and staining of CP explants

For adoptive transfer experiments, splenocytes of 2D2 mice were isolated and cultured under MCAM polarization conditions as described previously [22]. Briefly, cells were cultured for 5 days in RPMI (1640) supplemented with 10% heat-inactivated fetal calf serum (FCS), 1% penicillin-streptomycin, 1% L-glutamine, and 50 μM 2-mercaptoethanol (2-ME) in the presence of 10 μg/ml MOG_35–55_ peptide, 5 μg/ml anti-IFNγ (clone: XMG1.2), 0.5 μg/ml anti-IL-4 (clone: 11B11, both eBioscience), 5 ng/ml human TGFβ, and 20 ng/ml murine IL-23 (both R&D systems, Wiesbaden, Germany). CD4^+^ T cells were enriched by negative selection using MACS (Miltenyi Biotec, Bergisch Gladbach, Germany), labeled with 1 μM CellTracker™ Green CMFDA Dye (ThermoFisher, Waltham, MA, USA) and adoptively transferred into C57BL/6 recipient mice (2.4 × 10^6^ CD4^+^ T cells per mouse in 100 μl PBS) by i.v. injection. On days 2 and 5 after adoptive transfer choroid plexus explants were stained as follows. After transferring the explanted choroid plexus epithelia on glass slides, PBS + 0.3% tween20 was applied for 5 min followed by two washing steps in PBS for 5 min. CP explants were fixed applying PBS + 2.2% PFA + 2% glucose + 0.02% sodium azide for 20 min at RT, rinsed in PBS, and additionally fixed using 100% methanol for 6 min. After two subsequent washes in PBS for 5 min, unspecific binding was blocked by applying PBS + 0.3% tween20 + 10% normal goat serum for 30 min at RT and then stained with anti-laminin α4 (clone 377; 1:1000, rabbit anti mouse; [34]) in PBS + 0.3% tween20 for 2 h at RT. Subsequently, the stained CP explants were washed twice for 5 min in PBS and stained using a secondary goat anti-rabbit antibody (1:100; Alexa fluor 568; Life Technologies) for 1 h at RT. After another washing series in PBS, cell nuclei were stained using DAPI (1 μg/ml) in PBS for 5 min at RT, washed in PBS again, and mounted in fluorescent mounting medium (Dako).

### Cell culture and transmigration assays

Fibroblasts originated from primary human choroid plexus epithelial cells (labeled as HCPEpiC) that were purchased from ScienCell Research Laboratories (Carlsbad, CA, USA) and were cultured in Epithelial Cell Medium (EpiCM) supplemented with 2% fetal bovine serum (FBS; ProVitro, Berlin, Germany) on poly-L-lysine (2 μg/cm^2^; ScienCell Research Laboratories). Cultured cells were characterized by real-time quantitative PCR for expression of endothelial and epithelial markers as well as expression of laminin α4.

Primary human brain microvascular endothelial cells (HBMEC) were obtained from Pelobiotech GmbH (Planegg, Germany) and maintained in microvascular endothelial cell growth medium supplemented with FBS and endothelial cell growth factor (ECGF) (ProVitro) on fibronectin (Pelobiotech) as previously described [35]. Where indicated, HBMEC were treated with TNFα (500 U/ml; R&D systems) for 16 h. For transmigration assays, fibroblasts originating from HCPEpiC (1 × 10^5^ cells) or HBMEC (1 × 10^5^ cells) were seeded on poly-L-lysine or fibronectin-coated membranes of Transwell inserts (6.5 mm Transwells Pore Polyester Membrane Insert; pore size = 3.0 μm; Corning, Lowell, MA, USA) and grown to confluency. CD4^+^ T cells were purified from whole blood of healthy donors (HD) using RosetteSep CD4^+^ T cell enrichment cocktail (StemCell Technologies, Vancouver, BC, Canada). 2 × 10^5^ CD4^+^ cells were subsequently transferred to the endothelial cell layer and were allowed to migrate in RPMI medium supplemented with 2% B27 (Gibco, Eggenstein, Germany) as previously described [36]. Transmigrated cells were collected from the lower chamber after an incubation time of 4 h. CD4^+^ T cells from within the fibroblastic layer were obtained by incubating the cell layer for 10 min at 37 °C with Accutase (Sigma-Aldrich, Taufkirchen, Germany) and cells were detached by rinsing with PBS. For quantification, Calibrite beads (BD Biosciences, Heidelberg, Germany) were added prior to harvesting the cells. The relative cell numbers were determined by flow cytometry and migrated cells were stained with anti-MCAM (clone: P1H12; BD Pharmingen, Franklin Lakes, NJ, USA), anti-CD4 (clone: OKT4), anti-CD11a (clone: HI111), anti-CD49d (clone: 9F10), anti-CD62L (clone: DREG-56), and anti-CD45RA (clone: HI100) (all from BioLegend, Fell, Germany).

### Blocking antibodies for transmigration assays

The blocking antibodies anti-VLA4 (anti-CD49d/natalizumab, Tysabri; Biogen Idec, Cambridge, MA, USA) and anti-MCAM (clone PRX003; Prothena Biosciences Inc., Dublin, Ireland) were used at a concentration of 10 μg/ml. CD4^+^ lymphocytes were pre-incubated for 30 min with the antibodies diluted in PBS before washing out of the blocking antibodies and application to the transmigration assays.

### Real-time quantitative PCR

RNA was extracted from confluent monolayers of HCPEpiC-derived fibroblasts or HBMEC using TRIzol reagent (Invitrogen). cDNA was synthesized from 1 μg of total RNA using a standard protocol with random hexamer primers (ThermoScientifc). Real-time qPCR was performed in a StepOnePlus cycler (Applied Biosystems, Darmstadt, Germany) employing endogen control primers for 18sRNA as well as a TaqMan Gene Expression Assays specific for human laminin α4, cytokeratin 18, VE-cadherin, vimentin, or PECAM1 (Applied Biosystems, Darmstadt, Germany).

### Immunofluorescence staining

For immunofluorescence studies, 4-μm-thick formalin-fixed paraffin embedded (FFPE) murine brain sections were stained for MCAM (CD146, rabbit monoclonal IgG, clone: EPR3208; Abcam, Cambridge, UK). An amplification with TSA Plus Biotin kit (PerkinElmer, Waltham, USA) was performed according to the manufacturer’s instructions. As secondary antibodies goat-anti-rabbit-HRP (IgG; LifeTechnologies, Carlsbad, USA) and streptavidin AlexaFluor488 conjugate (Invitrogen, Carlsbad, USA) were used. Sections were mounted in Gold antifade containing 4′,6-diamidino-2-phenylindole (DAPI, Invitrogen). Slides were analyzed on a BioRevo microscope (BZ-9000, Keyence, Osaka, Japan) using the BZ-II Analyzer software.

Six-μm-thick cryosections of murine brain tissue were fixed with methanol and were stained for laminin α4 (clone 377b [37]), MECA32 antigen [38], and plectin (Progen, Heidelberg, Germany).

For human CNS tissue, autopsy material from subjects with MS was obtained from the Netherlands Brain Bank (NBB). Immunofluorescence stainings were performed on 10 μm cryosections and paraformaldehyde-fixed confluent monolayers of fibroblasts (derived from primary HCPEpiC) with a monoclonal anti-human laminin α4 antibody (clone 3D12 [39]) and an AlexaFluor488-labeled goat-anti-mouse secondary antibody. In case of the fibroblastic monolayers, an amplification step with TSA Plus Biotin kit (PerkinElmer, Waltham, MA, USA) was performed according to the manufacturer’s instructions. Tissues were analyzed using a Zeiss LSM700 confocal microscope. Images were analyzed using Volocity 6.3 software (ImproVision).

### Study approval

Studies on human samples were approved by the local ethics committee (University of Muenster: Ethik-Kommission der Ärztekammer Westfalen-Lippe und der Medizinischen Fakultät der Westfälischen Wilhelms-Universität, registration number: 2010-245-f-S). All experiments were performed according to the Declaration of Helsinki.

### Statistical analysis

All values are presented as mean ± standard error of the mean (SEM). Statistical significance was determined using Student’s *t* test in the case of normally distributed data; otherwise, a Mann-Whitney test was performed. For comparisons between data of CD4^+^ subpopulations from the same donor, the paired Student’s *t* test was used. The two-way ANOVA followed by Bonferroni post-test was applied for EAE experiments. *P* values were considered significant if < 0.05 in all cases. Statistical analyses were carried out using Prism (Version 5, GraphPad, San Diego, CA, USA).

## Results and discussion

### MCAM blockade delays EAE disease onset in T cell-specific integrin α4 knockout mice (CD4::*Itga4*^*−/−*^*)*

**Fig. 1:**
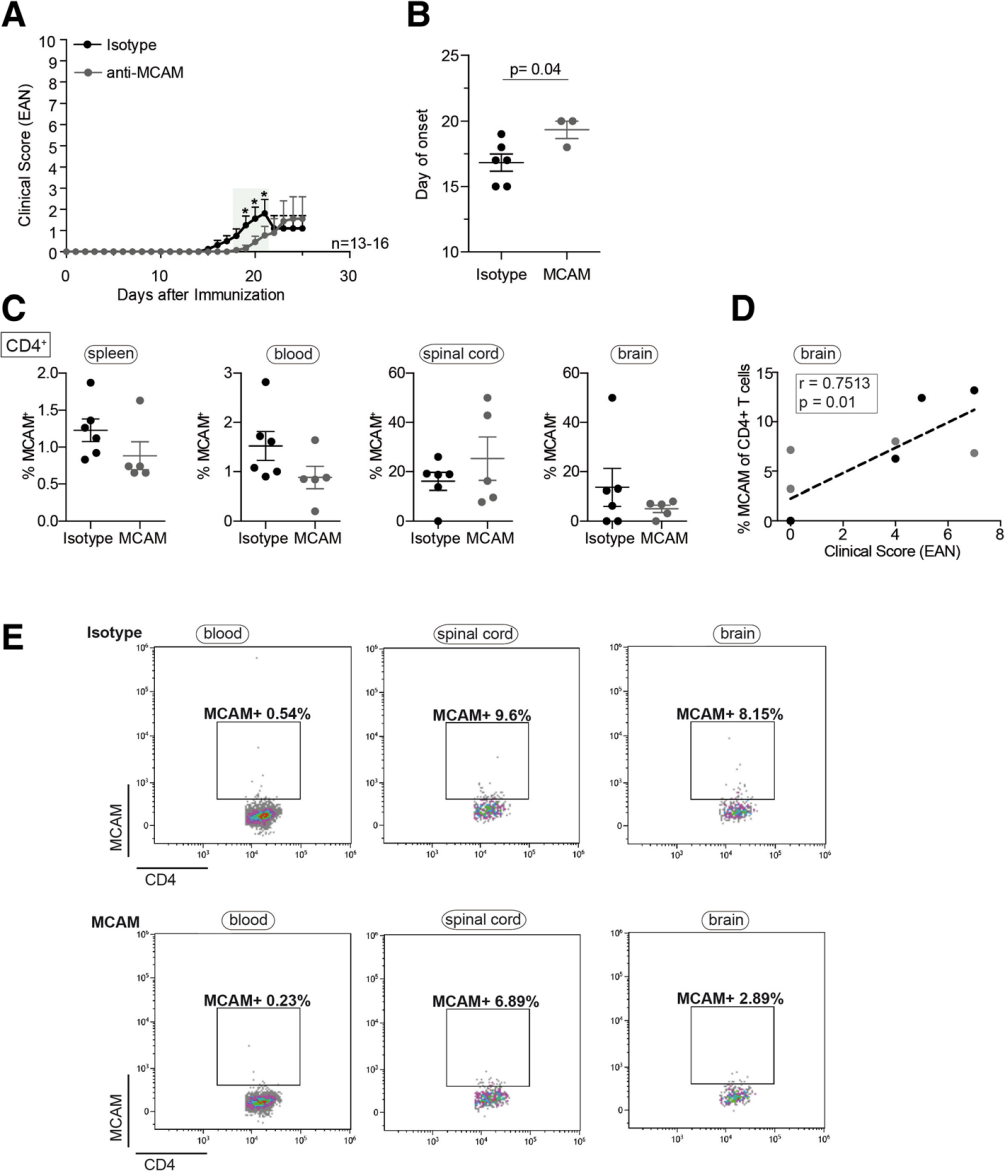
MCAM blockade delays EAE onset in T cell-specific *Itga4* knockout mice (CD4::*Itga4*^*−/−*^). **a** Development of active EAE in CD4::*Itga4*^*−/−*^ mice treated every other day after MOG_35–55_ immunization with anti-MCAM (clone 15) neutralizing antibody or isotype control antibody. Mean clinical EAN scores ± SEM of three independent experiments over time are shown; *P < 0.05; green highlighted areas: *P* < 0.1 (**b**) The average day of disease onset, defined as the first day with a score greater than or equal to 1, is shown as mean ± SEM. Percentages of MCAM CD4^+^ T cells (**c**) isolated from the spleen, blood, spinal cord, and brain of isotype control or anti-MCAM-treated mice were quantified by flow cytometry on day 22 post MOG_35–55_ immunization. Correlation analyses between the clinical score (EAN) and percentages of MCAM-expressing CD4^+^ T cells (**d**) in brains of isotype control (black dots) and anti-MCAM-treated mice (gray dots) on day 22 post immunization shows a positive correlation for CD4^+^T cells (Spearman *r* = 0.7513; *P* = 0.01). **e** Representative flow cytometric analyses of MCAM^+^ CD4^+^ T cells are shown for isotype control (upper panel) or anti-MCAM-treated mice (lower panel)

To investigate whether encephalitogenic CD4^+^ T cells without functionally active VLA-4 can employ MCAM to migrate into the CNS, we induced EAE in transgenic mice lacking α4-integrin expression on T cells (CD4::*Itga4*^−/−^) in the presence or absence of a function-blocking antibody to MCAM. MOG_35–55_ immunized CD4::*Itga4*^−/−^ mice developed a mild and clinically atypical EAE, as previously reported [12] (Fig. 1a). Application of anti-MCAM to CD4::*Itga4*^−/−^ mice every second day after MOG_35–55_ immunization commencing on day 0 resulted in a delay in disease onset (Fig. 1a, b and Table 1), but no change in disease severity. This suggests that in the absence of α4-integrin, MCAM blocking delays but does not completely prevent CD4^+^ T_H_17 entry into the CNS parenchyma. Former studies have shown that the chemokine receptor CCR6, which like MCAM is selectively upregulated in polarized murine T_H_17 cells [10, 22], is essential for CNS trafficking of T_H_17 cells to the CP. CCR6^+^ T_H_17^+^ cells are considered to represent the first wave of leukocyte infiltration required for the subsequent recruitment of other inflammatory cells (T_H_1 cells) across post-capillary venules and induction of disease symptoms [10]. T_H_17 cells are further able to access the CNS parenchyma independently of VLA-4 and MOG_35–55_ immunized mice lacking α4-integrin expression on T cells (CD4::*Itga4*^−/−^) exhibit marked immune cell infiltration within the plexus epithelium [12], indicating that in the absence of α4-integrin encephalitogenic T cells are recruited via the choroid plexus into the CNS. These findings and the observed delay in EAE onset upon MCAM blockade suggest that, besides CCR6, MCAM might be crucial for the initial infiltration of encephalitogenic T_H_17 cells via the CP. Further, our recent observation of a specific accumulation of MCAM-expressing lymphocytes in the CSF of patients with MS that are under long-term natalizumab therapy (VLA-4 blockade) [17] suggests also that MCAM-expressing lymphocytes migrate over the CP into the CNS.

**Table 1:** Summary of EAE incidence, onset, and maximum disease severity in different murine models of MS upon antibody mediated blocking of VLA-4, MCAM, or VLA-4 and MCAM

Mouse	Treatment	Incidence	Age (days)/day of onset	Cumulative score
Integrin α4^−/−^	Anti-MCAM	3/13 (23%)	19.3 ± 0.66	0.21 ± 0.12
Integrin α4^−/−^	Isotype (MCAM)	6/16 (38%)	16.8 ± 0.65	0.38 ± 0.14
Devic	Anti-VLA-4	1/11 (9%)	39 ± 0	0.01 ± 0.01
Devic	Isotype (VLA-4)	7/11 (64%)	33.7 ± 1.5	0.58 ± 0.16
Devic	Anti-MCAM	9/22 (41%)	34.3 ± 1.6	0.65 ± 0.19
Devic	Isotype (MCAM)	12/21 (57%)	36.1 ± 1.4	0.79 ± 0.19
Devic	Anti-VLA-4/MCAM	2/11 (18%)	35.5 ± 3.5	0.09 ± 0.07
Devic	Isotype (VLA-4/MCAM)	9/11 (82%)	30 ± 1.7	1.54 ± 0.09

The age/day of onset and the cumulative score are means ± SEM

Blockade of MCAM might possibly impact migration into the CNS via blocking of endothelial MCAM, as entry of encephalitogenic T cells into the brain is reduced in endothelial MCAM knockout mice [26]; however, flow cytometry performed on day 22 post immunization with an anti-mMCAM, antibody recognizing an epitope distinct from the therapeutic antibody, showed that administration of the MCAM neutralizing antibody resulted in lower expression of MCAM on natural killer (NK) cells (Additional file 1: Figure S1a) and CD4^+^ T cells both in the periphery and the CNS (quantification is shown in Fig. 1c and representative flow cytometric analysis in Fig. 1e), suggesting shedding or downregulation of MCAM on peripheral immune cells.

In mice, contrary to humans, MCAM is strongly expressed on NK cells [40], which might be a reason for the more pronounced downregulation in the percentages of MCAM^+^ NK cells compared to the slight effect on MCAM^+^ T cells. In CD4::*Itga4*^*−/−*^ mice-independent of the treatment, percentages of MCAM-expressing CD4^+^ cells in the brain correlated with the clinical score (Spearman *r* = 0.7513; *P* = 0.01) (Fig. 1d), whereas MCAM-expressing NK cell proportions in the brain did not correlate with clinical score (Spearman *r* = − 0.4427; *P* = 0.2) (Additional file 1: Figure S1b), indicating that the severity of EAE depends on the amount of MCAM^+^ CD4^+^ T cells in the brain. As CD4^+^ T cells are the primary pathogenic T cells in EAE [41], this suggests that antibody blocking of MCAM affects either MCAM-expressing T_H_17 cell numbers in the periphery or their entry into the CNS and, thereby, EAE induction.

### Blockade of VLA-4 and MCAM in a spontaneous mouse model of MS (“Devic” mice)

**Fig. 2:**
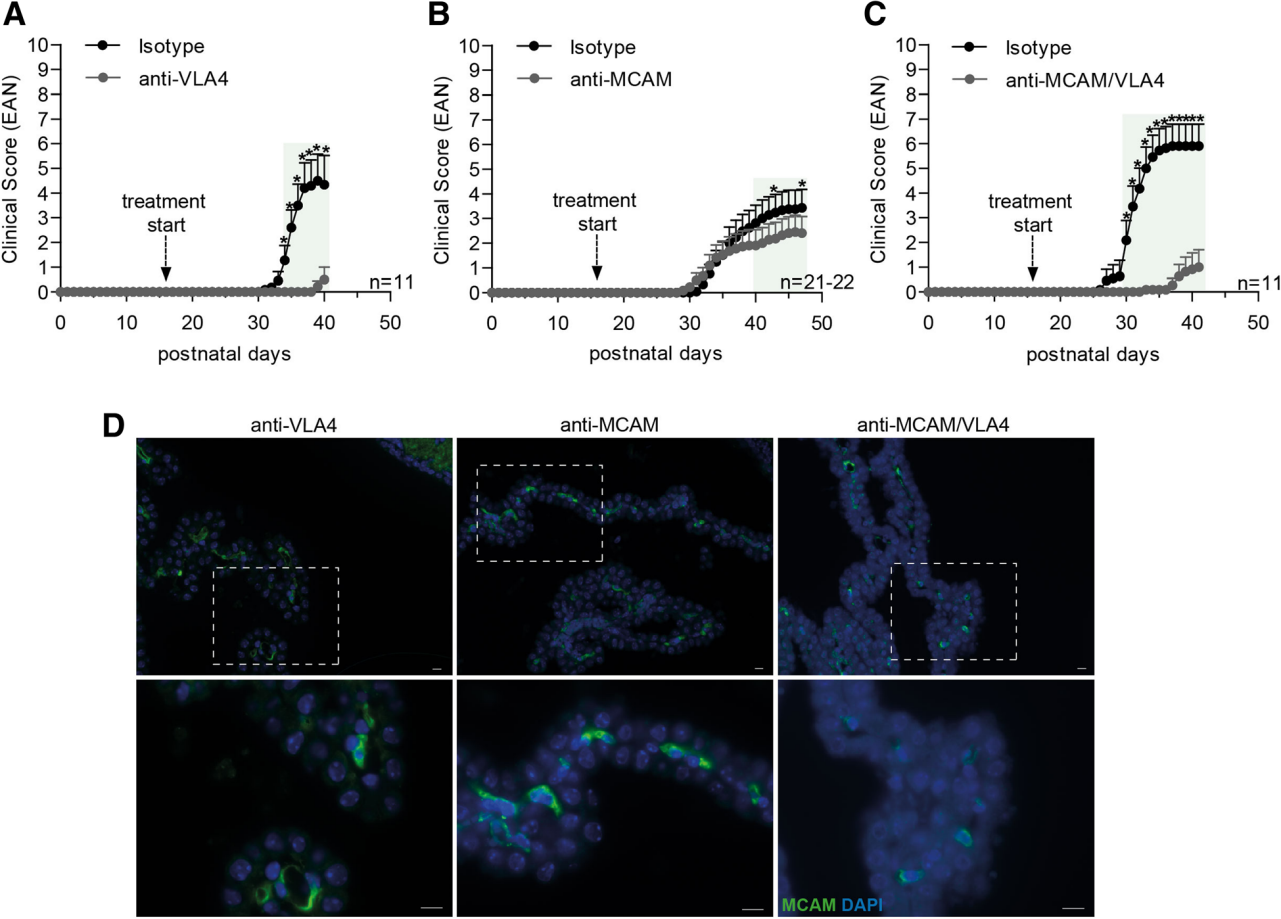
MCAM and VLA4 blockade in a model of spontaneous EAE. Development of spontaneous EAE-like disease in Devic mice that were either treated with (**a**) anti-VLA4 (clone PS/2), (**b**) anti-MCAM (clone 15), (**c**) anti-MCAM and anti-VLA4 antibodies, or the respective isotype control antibodies. Mice were treated every second day from day 16 after birth. Littermates were used for the treatment with either the respective blocking antibody or the isotype control. Mean clinical EAN scores ± SEM of three independent experiments over time are shown; *P < 0.05; green highlighted areas: *P* < 0.1. (**d**) Immunofluorescence staining of MCAM (green) in choroid plexus tissue obtained from Devic mice treated with anti-VLA-4, anti-MCAM, or anti-MCAM/VLA-4 neutralizing antibodies. Nuclear staining (DAPI) is shown in blue. Scale bars are 10 μm

To assess whether anti-MCAM effects are specific to T_H_17 versus T_H_1-induced inflammation, we utilized a spontaneous mouse model of MS (“Devic” mice), which shows largely optic nerve and spinal cord leukocyte infiltration and is mainly characterized by a T_H_1 cytokine profile [31, 32, 42]. Devic mice are a double-transgenic mouse strain, expressing T and B cell receptors that recognize the same autoantigen (MOG), in which around 50% of the double-transgenic offspring spontaneously develop a paralytic disease reminiscent of a subset of neuromyelitis optica patients. Devic mice were treated every second day from day 16 after birth with either anti-VLA4, anti-MCAM, or both antibodies; control mice were treated with the appropriate isotype control antibodies. VLA-4 blockade largely protected mice from the development of paralytic disease (Fig. 2a and Table 1), whereas blockade of MCAM resulted in reduced disease incidence and severity (Fig. 2b and Table 1). Blockade of both VLA-4 and MCAM did not show an additive effect compared to VLA-4 blockade alone (Fig. 2c and Table 1). These results are consistent with the reported high expression of VLA-4 on T_H_1 cells and the comparatively low VLA-4 levels on MCAM^high^ T_H_17 cells [12, 22]. In addition, the data suggest that the effects of anti-MCAM treatment are most pronounced in T_H_17-mediated EAE and therefore affect T_H_17 trafficking to the brain. Previous experiments employing murine models of MS with encephalitogenic T cells expressing VLA-4 showed that administration of a MCAM blocking antibody affected disease progression during remission [22–24]. However, mice with Devic’s disease normally do not enter remission, which could explain why we did not observe effects of the MCAM treatment in the late stage of the disease.

In order to corroborate the influence of MCAM in CNS inflammation in the Devic model, we performed histological analyses of the CP tissue to investigate whether MCAM blockade might impact migration into the CNS via blocking of endothelial MCAM. However, endothelial MCAM expressing cells could easily be detected in the CP tissue of mice treated with anti-MCAM blocking antibody or with a synergistic blockade of MCAM and VLA-4 (Fig. 2d), suggesting that this anti-MCAM neutralizing antibody does not have any impact on endothelial MCAM and acts on peripheral immune cells.

**Fig. 3:**
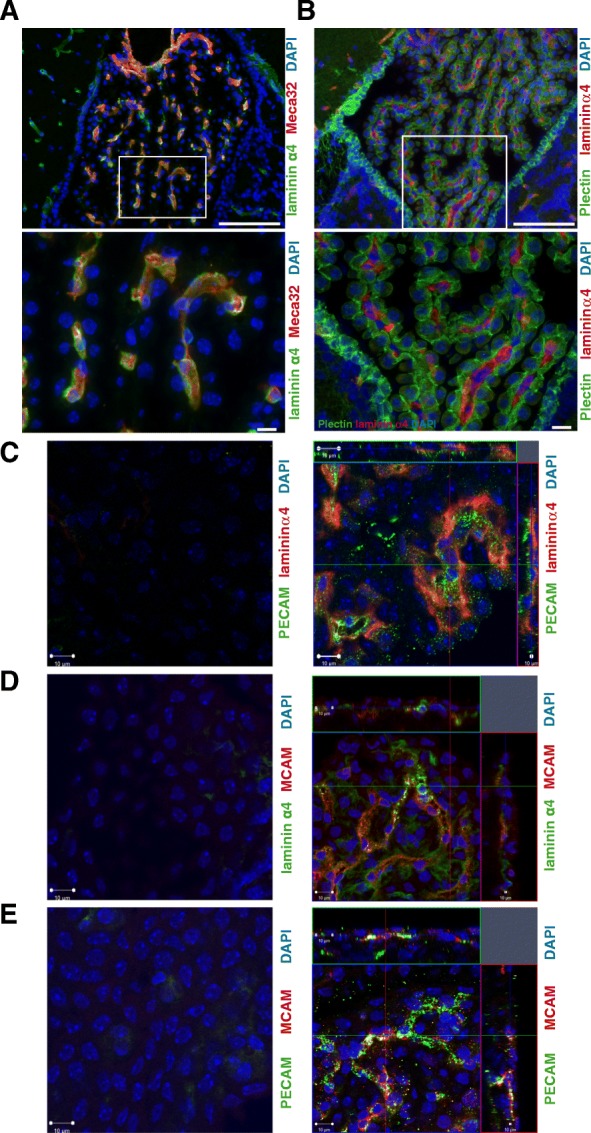
Laminin α4 localization to choroid plexus endothelial basement membranes. C57BL/6 mouse choroid plexus samples immunofluorescently stained for (**a**) laminin α4 (green) and MECA32 antigen as an endothelial cell marker (red) or (**b**) laminin α4 (red) and plectin to mark choroid epithelium (green), demonstrating colocalization of laminin α4 with the MECA32^+^ blood vessels located beneath the epithelial layers. Cell nuclei are counterstained with DAPI (blue). Higher magnifications of the boxed areas are shown in the lower panels. Bars are 100 μm (upper panels) and 10 μm (lower panels), respectively. **c–e** Immunofluorescence staining of choroid plexus explants of C57BL/6 mice for (**c**) laminin α4 (red) and PECAM (green), (**d**) laminin α4 (green) and MCAM (red), and (**e**) PECAM (green) and MCAM (red). Nuclear staining (DAPI) is shown in blue, and left panels represent control stainings. Scale bars: 10 μm

To assess the relevance of MCAM expression on T cells for their transmigration across the CP, we performed histological stainings of laminin α4, the proposed major ligand of MCAM, in murine CP tissue. Double staining of CP tissue from healthy control mice for endothelial or epithelial markers together with the MCAM ligand laminin α4 revealed laminin α4 staining exclusively in association with the MECA32^+^ endothelial cells of the CP (Fig. 3a) located between the plectin^+^ epithelial cell layers (Fig. 3b) [43, 44]. Blockade of MCAM might therefore ameliorate CNS inflammation by preventing migration of MCAM expressing cells across the endothelial CP layer on their way into the CNS.

In a proof-of-concept experiment, we transferred fluorescently labeled myelin oligodendrocyte glycoprotein-specific TCR-transgenic T cells (2D2 T cells), differentiated in vitro with a protocol that induces high percentages of MCAM expressing cells [22], into C57BL/6 recipient mice. Immunofluorescence staining of cortical explants at days 2 and 5 after transfer revealed individual MCAM expressing CD4^+^ T cells in the CP, providing first evidence that MCAM-expressing T cells can migrate through the CP and potentially into the CNS (Representative images are shown in Additional file 2: Figure S2; numbers of transferred cells, that were detected in the CP: mouse 1: 23; mouse 2: 19; mouse 3: 17). Staining of laminin α4 in cortical explants confirmed further the localization of the MCAM ligand to endothelial basement membranes of the CP (Fig. 3c–e).

### Enhanced migration of human MCAM-expressing T cells across endothelial and extracellular matrix barriers is dependent on MCAM-laminin 411 interactions

**Fig. 4:**
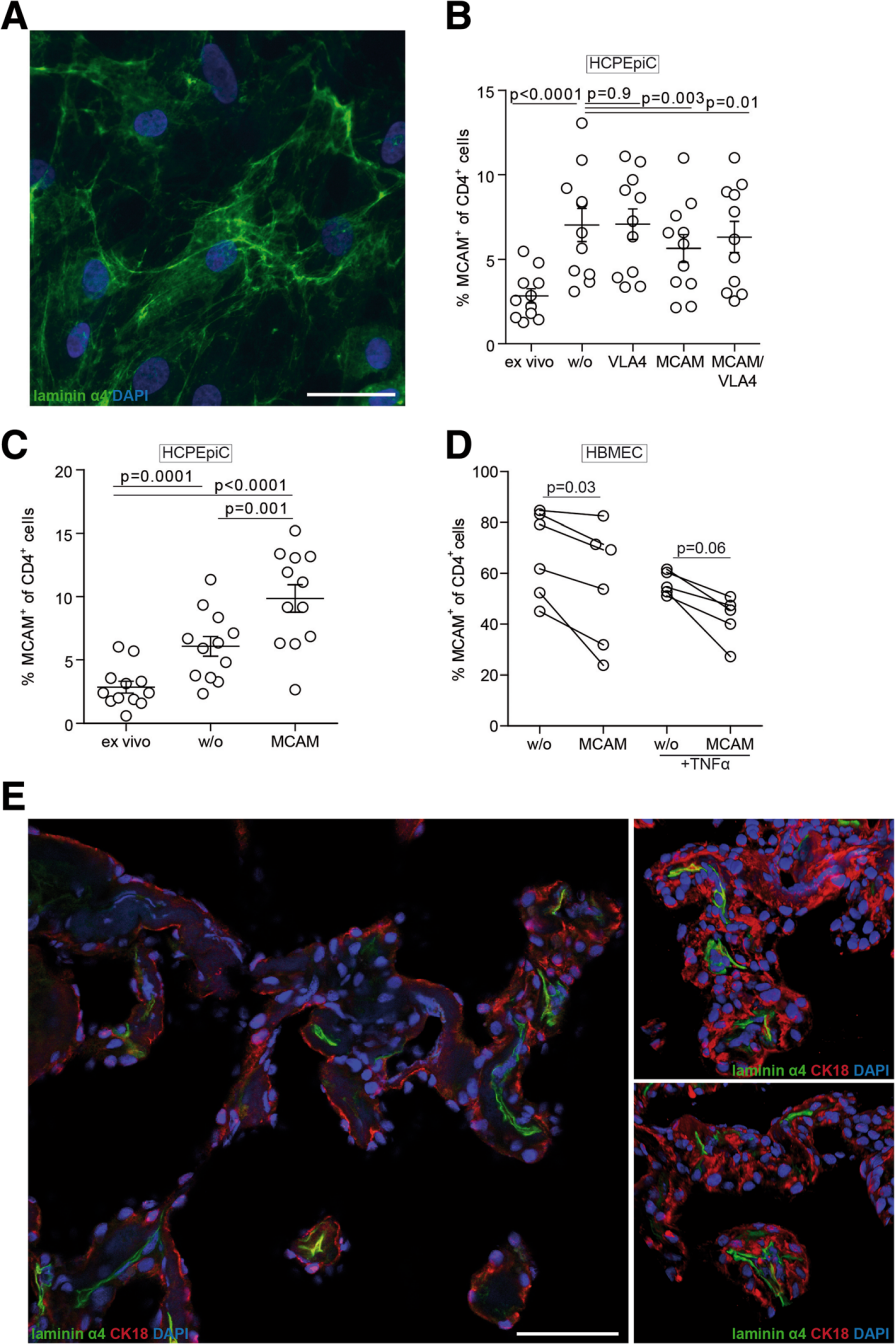
MCAM-laminin α4 interactions mediate lymphocyte transmigration across human endothelial and fibroblastic layers. **a** Immunofluorescence staining of cultured fibroblasts derived from primary human choroid plexus cells (HCPEpiC) with anti-laminin α4 (green); cell nuclei are counterstained with DAPI (blue). Bar is 50 μm. **b** In vitro transmigration of CD4^+^ lymphocytes through the fibroblastic plus connective tissue layer in a modified Boyden chamber assay. Shown are the percentages of MCAM^+^ CD4^+^ T cells before inclusion in the transmigration assay (ex vivo) and after transmigration without (w/o) blocking antibody or with VLA4, MCAM, or MCAM and VLA4 blocking antibodies. Data are from four independent experiments. **c** Percentages of MCAM^+^ CD4^+^ T cells retained in the HCPEpiC layer in the absence (w/o) or presence of MCAM blocking antibody compared to the original sample (ex vivo). Data are from four independent experiments. **d** Percentages of MCAM^+^ CD4^+^ T cells before and after transmigration of a HBMEC layer without (w/o) blocking antibody or with MCAM blocking antibody, under non-inflamed or inflammatory conditions (+TNFα). Data are from three independent experiments. **e** Laminin α4 (green) staining of human choroid plexus tissue from MS CNS samples. Nuclear staining (DAPI) is shown in blue, and the epithelial cell marker cytokeratin 18 (CK18) is shown in red. Bar is 50 μm

To further investigate whether MCAM has an effect on lymphocyte migration over CP endothelial barriers, we performed in vitro T cell transmigration assays using primary human brain-derived microvascular endothelial cells (HBMECs) and choroid plexus fibroblasts (labeled as “HCPEpiC”) (characterization of the cells is shown in Additional file 3: Figure S3). We have previously shown that laminin α4 can be expressed by fibroblasts in different tissues but is deposited in subjacent endothelial basement membranes [27] and that it is also expressed by cultured HBMECs [17]. Immunofluorescent staining confirmed that laminin α4 is present on cultured HCPEpiC-derived fibroblasts in vitro (Fig. 4a), and MCAM blockade indeed restricted transmigration of MCAM-expressing T cells (Fig. 4b). The proportion of MCAM^+^ cells among CD4^+^ T cells was enriched in the fraction of cells that transmigrated the HCPEpiC-derived fibroblasts layer, compared to the initial (ex vivo) CD4^+^ T cell fraction, but was significantly reduced when the CD4^+^ lymphocytes were pre-incubated with a MCAM blocking antibody, either alone or together with anti-VLA-4. Moreover, analysis of fibroblastic layer following the transmigration period, revealed enrichment of MCAM^+^ CD4^+^ T cells within the cell layer (Fig. 4c), suggesting adherence of MCAM^+^ lymphocytes to laminin α4 in the deposited extracellular matrix during their migration through the cell layer. MCAM blockade led to marked accumulation of MCAM^+^ CD4^+^ T cells within the cell layer (Fig. 4c), suggesting that the cells can still adhere to the fibroblastic cell layer, but require MCAM-mediated processes for efficient transmigration. This was substantiated by the results of transmigration over both TNF-α inflamed or non-inflamed HBMECs, which also revealed reduced MCAM^+^ CD4^+^ T cell transmigration over an endothelial layer in presence of a MCAM blocking antibody (Fig. 4d).

To determine whether these in vitro findings are also relevant to the human in vivo system, we stained human CP tissue from one MS patient for laminin α4. We detected high laminin α4 expression in association with the endothelial layer of the CP, whereas no laminin α4 staining was detectable on the epithelial layer (Fig. 4e). The pattern of laminin α4 localization, therefore, supports the hypothesis that MCAM-laminin α4 interactions facilitate MCAM^+^CD4^+^ T cell penetration of the CP endothelial layer and thereby entry into the CNS during neuro-inflammation.

The differential effects of blocking VLA-4 and/or MCAM in active EAE and in the spontaneous EAE model suggest that targeting VLA-4 restricts most encephalitogenic T cells from migrating into the CNS, while blockade of MCAM more specifically inhibits T_H_17 cell migration into the CNS via the CP endothelium. Effects of MCAM blockade are particularly pronounced in murine models of MS that depend on CNS migration across the CP, such as in the case of mice lacking α4-integrin expression on T cell. This is in accordance with our previous observation of a specific accumulation of MCAM^+^ CD4^+^ T cells in the CSF of MS patients with long-term Natalizumab therapy (VLA-4 blockade) [17], suggesting that MCAM^+^ CD4^+^ lymphocytes preferentially migrate over the CP into the CNS. This may be further enhanced by the high expression of the chemokine receptor CCR6, a characteristic marker of the encephalitogenic T_H_17 cells [10], which binds to CCL20 expressed on the choroid plexus epithelium in mice [45] and confirmed here for human tissue (see Additional file 4: Figure S4).

Leukocytes that are recruited to the CP and thereby into the CNS have to migrate through the endothelium and underlying basement membrane before they can migrate through the epithelial barrier into the CSF. Here, we show that MCAM is required for migration of an encephalitogenic MCAM^+^ CD4^+^ subpopulation of T cells into the CP*,* as cells without MCAM showed reduced migration across both endothelial and fibroblastic laminin 411 expressing layers in vitro and reduced CNS infiltration in vivo.

Accordingly, laminin α4 was localized to basement membranes underlying the choroidal endothelium, but not choroidal epithelium, suggesting that MCAM-laminin 411 interactions mediate the initial choroidal endothelium crossing in vivo. The differential expression of laminin isoforms as a “doorway” for leukocyte migration across endothelial of post-capillary venules in the CNS parenchyma has previously been reported [34, 46, 47] and, therefore, a similar concept might also apply for the choroidal endothelium.

Importantly, by interfering with migration over the CP endothelium, MCAM blockade might have a specific effect on disease initiation. Others have previously reported the CP as the site of T cell entry into the CNS during immune surveillance and at first stages of neuro-inflammation [10].

However, besides the CP, there are other entry routes into the CNS, such as through the circumventricular organs or through the leptomeninges, both of which are widely considered to be important in acute inflammation and perpetuation of CNS disease. In a rat EAE model, the leptomeninges was recently identified as an important checkpoint for T cell infiltration of the CNS, where effector T cells can enter the CSF and eventually invade the parenchyma [48]. These alternative entry routes into the CNS, which were not assessed in this study, might be the reason why a complete prevention of autoimmune CNS inflammation upon MCAM blockade in the absence of VLA-4 was not be observed.

## Conclusions

Our results indicate that targeting MCAM results in an overall lower, but more specific clinical improvement compared to targeting VLA-4 and seems to affect mainly T_H_17 cells. This suggests that therapeutic blocking of MCAM might be advantageous over VLA-4 blocking, in terms of the balance of blocking pathogenic cells versus immune surveillance mechanisms and may, therefore, have fewer adverse effects. Hence, in case of a clear T_H_17 pathology, blocking of MCAM, instead or in addition, to VLA-4 or basic therapies such as anti-interferon treatment might represent a new therapeutic avenue.

## Additional files

Additional file 1: **Figure S1.** MCAM expression on NK cells does not correlate with clinical score. Percentages of MCAM-expressing NK1.1^+^ cells (**a**) isolated from the spleen, blood, spinal cord, and brain of isotype control or anti-MCAM-treated mice were quantified by flow cytometry on day 22 post MOG_35–55_ immunization. Correlation analyses between the clinical score (EAN) and percentages of MCAM-expressing NK1.1^+^ cells (**b**) in brains of isotype control (black dots) and anti-MCAM-treated mice (gray dots) on day 22 post immunization show no correlation for NK1.1^+^ cells (Spearman *r* = − 0.4427; n.s.). (JPG 1119 kb)

Additional file 2: **Figure S2.** MCAM-expressing CD4^+^ T cell migrate through the choroid plexus into the CNS. Representative images of adoptively transferred myelin oligodendrocyte glycoprotein-specific TCR-transgenic T cells (2D2 T cells) in whole-mount murine choroid plexus samples. 2D2 T cells were differentiated in vitro for 5 days under MCAM polarizing conditions (MOG_35–55_, TGFβ, IL-23), CMFDA-labeled, and transferred by i.v. injection to three C57BL/6 recipient mice. Immunofluorescence staining of choroid plexus explants on day 2 (mouse 1; **a**) and day 5 (mouse 2, 3; **b, c**) after adoptive transfer for CMFDA^+^ CD4^+^ T cells (green), and nuclei (blue; DAPI). Scale bars: 10 μm. (TIF 913 kb)

Additional file 3: **Figure S3.** Characterization of human brain-derived microvascular endothelial cells and fibroblasts derived from human choroid plexus cells. mRNA levels of laminin α4, cytokeratin 18 (CK18), PECAM1, VE-cadherin, and vimentin in primary human brain-derived microvascular endothelial cells (HBMEC) and fibroblasts originated from primary human choroid plexus epithelial cells (labeled as HCPEpiC) were quantified by real-time PCR, revealing the lack of epithelial markers by HCPEpiC and confirming their fibroblastic nature. n.d. = not detected (TIF 4578 kb)

Additional file 4: **Figure S4.** CCL20 localization on human choroid plexus tissue. CCL20 staining (green) on human choroid plexus epithelium in control CNS tissue samples. Nuclear staining (DAPI) is shown in blue. Scale bars = 100 μm. (TIF 6336 kb)

## Abbreviations

BBB: Blood-brain barrier
CNS: Central nervous system
CP: Choroid plexus
CSF: Cerebrospinal fluid
EAE: Experimental autoimmune encephalomyelitis
HBMECs: Human brain-derived microvascular endothelial cells
HCPEpiC: Human choroid plexus epithelial cells
ICAM-1: Intercellular adhesion molecule-1
LFA-1: Leukocyte function-associated molecule-1
MCAM: Melanoma cell adhesion molecule
MOG: Myelin oligocendrocyte glycoprotein
MS: Multiple sclerosis
NK: Natural killer
T_H_: T helper
VCAM-1: Vascular cell adhesion molecule-1
VLA-4: Very late antigen-4

## References

[1] Alberio L, Dale GL (1999). Review article: platelet-collagen interactions: membrane receptors and intracellular signalling pathways. Eur J Clin Investig.

[2] Nylander A, Hafler DA (2012). Multiple sclerosis. J Clin Invest.

[3] Dendrou CA, Fugger L, Friese MA (2015). Immunopathology of multiple sclerosis. Nat Rev Immunol.

[4] Frohman EM, Racke MK, Raine CS (2006). Multiple sclerosis--the plaque and its pathogenesis. N Engl J Med.

[5] Polman CH, O'Connor PW, Havrdova E, Hutchinson M, Kappos L, Miller DH, Phillips JT, Lublin FD, Giovannoni G, Wajgt A (2006). A randomized, placebo-controlled trial of natalizumab for relapsing multiple sclerosis. N Engl J Med.

[6] Yednock TA, Cannon C, Fritz LC, Sanchez-Madrid F, Steinman L, Karin N (1992). Prevention of experimental autoimmune encephalomyelitis by antibodies against alpha 4 beta 1 integrin. Nature.

[7] Steiner O, Coisne C, Cecchelli R, Boscacci R, Deutsch U, Engelhardt B, Lyck R (2010). Differential roles for endothelial ICAM-1, ICAM-2, and VCAM-1 in shear-resistant T cell arrest, polarization, and directed crawling on blood-brain barrier endothelium. J Immunol.

[8] Klotz L, Burgdorf S, Dani I, Saijo K, Flossdorf J, Hucke S, Alferink J, Nowak N, Beyer M, Mayer G (2009). The nuclear receptor PPAR gamma selectively inhibits Th17 differentiation in a T cell-intrinsic fashion and suppresses CNS autoimmunity. J Exp Med.

[9] Korn T, Bettelli E, Oukka M, Kuchroo VK (2009). IL-17 and Th17 cells. Annu Rev Immunol.

[10] Reboldi A, Coisne C, Baumjohann D, Benvenuto F, Bottinelli D, Lira S, Uccelli A, Lanzavecchia A, Engelhardt B, Sallusto F (2009). C-C chemokine receptor 6-regulated entry of TH-17 cells into the CNS through the choroid plexus is required for the initiation of EAE. Nat Immunol.

[11] Glatigny S, Duhen R, Arbelaez C, Kumari S, Bettelli E (2015). Integrin alpha L controls the homing of regulatory T cells during CNS autoimmunity in the absence of integrin alpha 4. Sci Rep.

[12] Rothhammer V, Heink S, Petermann F, Srivastava R, Claussen MC, Hemmer B, Korn T (2011). Th17 lymphocytes traffic to the central nervous system independently of alpha4 integrin expression during EAE. J Exp Med.

[13] Rothhammer V, Muschaweckh A, Gasteiger G, Petermann F, Heink S, Busch DH, Heikenwalder M, Hemmer B, Drexler I, Korn T (2014). alpha4-integrins control viral meningoencephalitis through differential recruitment of T helper cell subsets. Acta Neuropathol Commun.

[14] Engelhardt B, Ransohoff RM (2012). Capture, crawl, cross: the T cell code to breach the blood-brain barriers. Trends Immunol.

[15] Engelhardt B, Sorokin L (2009). The blood-brain and the blood-cerebrospinal fluid barriers: function and dysfunction. Semin Immunopathol.

[16] Wilson EH, Weninger W, Hunter CA (2010). Trafficking of immune cells in the central nervous system. J Clin Invest.

[17] Schneider-Hohendorf T, Rossaint J, Mohan H, Boning D, Breuer J, Kuhlmann T, Gross CC, Flanagan K, Sorokin L, Vestweber D (2014). VLA-4 blockade promotes differential routes into human CNS involving PSGL-1 rolling of T cells and MCAM-adhesion of TH17 cells. J Exp Med.

[18] Dagur PK, McCoy JP (2015). Endothelial-binding, proinflammatory T cells identified by MCAM (CD146) expression: characterization and role in human autoimmune diseases. Autoimmun Rev.

[19] Brucklacher-Waldert V, Stuerner K, Kolster M, Wolthausen J, Tolosa E (2009). Phenotypical and functional characterization of T helper 17 cells in multiple sclerosis. Brain.

[20] Dagur PK, Biancotto A, Stansky E, Sen HN, Nussenblatt RB, McCoy JP (2014). Secretion of interleukin-17 by CD8+ T cells expressing CD146 (MCAM). Clin Immunol.

[21] Dagur PK, Biancotto A, Wei L, Sen HN, Yao M, Strober W, Nussenblatt RB, McCoy JP (2011). MCAM-expressing CD4(+) T cells in peripheral blood secrete IL-17A and are significantly elevated in inflammatory autoimmune diseases. J Autoimmun.

[22] Flanagan K, Fitzgerald K, Baker J, Regnstrom K, Gardai S, Bard F, Mocci S, Seto P, You M, Larochelle C (2012). Laminin-411 is a vascular ligand for MCAM and facilitates TH17 cell entry into the CNS. PLoS One.

[23] Larochelle C, Cayrol R, Kebir H, Alvarez JI, Lecuyer MA, Ifergan I, Viel E, Bourbonniere L, Beauseigle D, Terouz S (2012). Melanoma cell adhesion molecule identifies encephalitogenic T lymphocytes and promotes their recruitment to the central nervous system. Brain.

[24] Larochelle C, Lecuyer MA, Alvarez JI, Charabati M, Saint-Laurent O, Ghannam S, Kebir H, Flanagan K, Yednock T, Duquette P (2015). Melanoma cell adhesion molecule-positive CD8 T lymphocytes mediate central nervous system inflammation. Ann Neurol.

[25] Larochelle C, Alvarez JI, Prat A (2011). How do immune cells overcome the blood-brain barrier in multiple sclerosis?. FEBS Lett.

[26] Duan H, Xing S, Luo Y, Feng L, Gramaglia I, Zhang Y, Lu D, Zeng Q, Fan K, Feng J (2013). Targeting endothelial CD146 attenuates neuroinflammation by limiting lymphocyte extravasation to the CNS. Sci Rep.

[27] Frieser M, Nockel H, Pausch F, Roder C, Hahn A, Deutzmann R, Sorokin LM (1997). Cloning of the mouse laminin alpha 4 cDNA. Expression in a subset of endothelium. Eur J Biochem.

[28] Scott LM, Priestley GV, Papayannopoulou T (2003). Deletion of alpha4 integrins from adult hematopoietic cells reveals roles in homeostasis, regeneration, and homing. Mol Cell Biol.

[29] Bettelli E, Pagany M, Weiner HL, Linington C, Sobel RA, Kuchroo VK (2003). Myelin oligodendrocyte glycoprotein-specific T cell receptor transgenic mice develop spontaneous autoimmune optic neuritis. J Exp Med.

[30] Litzenburger T, Fassler R, Bauer J, Lassmann H, Linington C, Wekerle H, Iglesias A (1998). B lymphocytes producing demyelinating autoantibodies: development and function in gene-targeted transgenic mice. J Exp Med.

[31] Bettelli E, Baeten D, Jager A, Sobel RA, Kuchroo VK (2006). Myelin oligodendrocyte glycoprotein-specific T and B cells cooperate to induce a Devic-like disease in mice. J Clin Invest.

[32] Krishnamoorthy G, Lassmann H, Wekerle H, Holz A (2006). Spontaneous opticospinal encephalomyelitis in a double-transgenic mouse model of autoimmune T cell/B cell cooperation. J Clin Invest.

[33] Breuer J, Schwab N, Schneider-Hohendorf T, Marziniak M, Mohan H, Bhatia U, Gross CC, Clausen BE, Weishaupt C, Luger TA (2014). Ultraviolet B light attenuates the systemic immune response in central nervous system autoimmunity. Ann Neurol.

[34] Sixt M, Engelhardt B, Pausch F, Hallmann R, Wendler O, Sorokin LM (2001). Endothelial cell laminin isoforms, laminins 8 and 10, play decisive roles in T cell recruitment across the blood-brain barrier in experimental autoimmune encephalomyelitis. J Cell Biol.

[35] Breuer J, Herich S, Schneider-Hohendorf T, Chasan AI, Wettschureck N, Gross CC, Loser K, Zarbock A, Roth J, Klotz L, et al. Dual action by fumaric acid esters synergistically reduces adhesion to human endothelium. Mult Scler. 2017:1352458517735189. 10.1177/1352458517735189. 10.1177/1352458517735189 28984166

[36] Schneider-Hohendorf T, Stenner MP, Weidenfeller C, Zozulya AL, Simon OJ, Schwab N, Wiendl H (2010). Regulatory T cells exhibit enhanced migratory characteristics, a feature impaired in patients with multiple sclerosis. Eur J Immunol.

[37] Ringelmann B, Roder C, Hallmann R, Maley M, Davies M, Grounds M, Sorokin L (1999). Expression of laminin alpha1, alpha2, alpha4, and alpha5 chains, fibronectin, and tenascin-C in skeletal muscle of dystrophic 129ReJ dy/dy mice. Exp Cell Res.

[38] Hallmann R, Mayer DN, Berg EL, Broermann R, Butcher EC (1995). Novel mouse endothelial cell surface marker is suppressed during differentiation of the blood brain barrier. Dev Dyn.

[39] Korpos E, Kadri N, Kappelhoff R, Wegner J, Overall CM, Weber E, Holmberg D, Cardell S, Sorokin L (2013). The peri-islet basement membrane, a barrier to infiltrating leukocytes in type 1 diabetes in mouse and human. Diabetes.

[40] Despoix N, Walzer T, Jouve N, Blot-Chabaud M, Bardin N, Paul P, Lyonnet L, Vivier E, Dignat-George F, Vely F (2008). Mouse CD146/MCAM is a marker of natural killer cell maturation. Eur J Immunol.

[41] Stromnes IM, Goverman JM (2006). Active induction of experimental allergic encephalomyelitis. Nat Protoc.

[42] Ransohoff RM (2006). A mighty mouse: building a better model of multiple sclerosis. J Clin Invest.

[43] Agrawal S, Anderson P, Durbeej M, van Rooijen N, Ivars F, Opdenakker G, Sorokin LM (2006). Dystroglycan is selectively cleaved at the parenchymal basement membrane at sites of leukocyte extravasation in experimental autoimmune encephalomyelitis. J Exp Med.

[44] Song J, Wu C, Korpos E, Zhang X, Agrawal SM, Wang Y, Faber C, Schafers M, Korner H, Opdenakker G (2015). Focal MMP-2 and MMP-9 activity at the blood-brain barrier promotes chemokine-induced leukocyte migration. Cell Rep.

[45] Zhang X, Wu C, Song J, Gotte M, Sorokin L (2013). Syndecan-1, a cell surface proteoglycan, negatively regulates initial leukocyte recruitment to the brain across the choroid plexus in murine experimental autoimmune encephalomyelitis. J Immunol.

[46] Song J, Zhang X, Buscher K, Wang Y, Wang H, Di Russo J, Li L, Lutke-Enking S, Zarbock A, Stadtmann A (2017). Endothelial basement membrane laminin 511 contributes to endothelial junctional tightness and thereby inhibits leukocyte transmigration. Cell Rep.

[47] Wu C, Ivars F, Anderson P, Hallmann R, Vestweber D, Nilsson P, Robenek H, Tryggvason K, Song J, Korpos E (2009). Endothelial basement membrane laminin alpha5 selectively inhibits T lymphocyte extravasation into the brain. Nat Med.

[48] Schlager C, Korner H, Krueger M, Vidoli S, Haberl M, Mielke D, Brylla E, Issekutz T, Cabanas C, Nelson PJ (2016). Effector T-cell trafficking between the leptomeninges and the cerebrospinal fluid. Nature.

