# Supplementary figures and images for: Blockade of MCAM/CD146 impedes CNS infiltration of T cells over the choroid plexus
Source: J Neuroinflammation. 2018 Aug 22;15:236. doi: 10.1186/s12974-018-1276-4 (PMC6106934; doi:10.1186/s12974-018-1276-4)

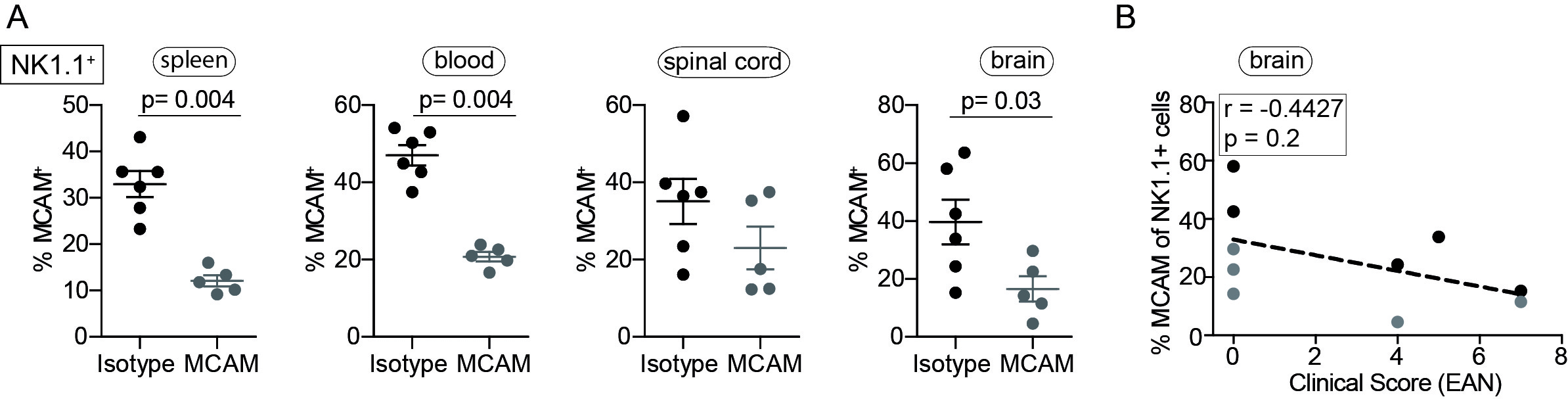

Supplement: Supplementary file 1 — Figure S1. MCAM expression on NK cells does not correlate with clinical score. Percentages of MCAM-expressing NK1.1+ cells (a) isolated from the spleen, blood, spinal cord, and brain of isotype control or anti-MCAM-treated mice were quantified by flow cytometry on day 22 post MOG35–55 immunization. Correlation analyses between the clinical score (EAN) and percentages of MCAM-expressing NK1.1+ cells (b) in brains of isotype control (black dots) and anti-MCAM-treated mice (gray dots) on day 22 post immunization show no correlation for NK1.1+ cells (Spearman r = − 0.4427; n.s.). (JPG 1119 kb) [file 12974_2018_1276_MOESM1_ESM.jpg]

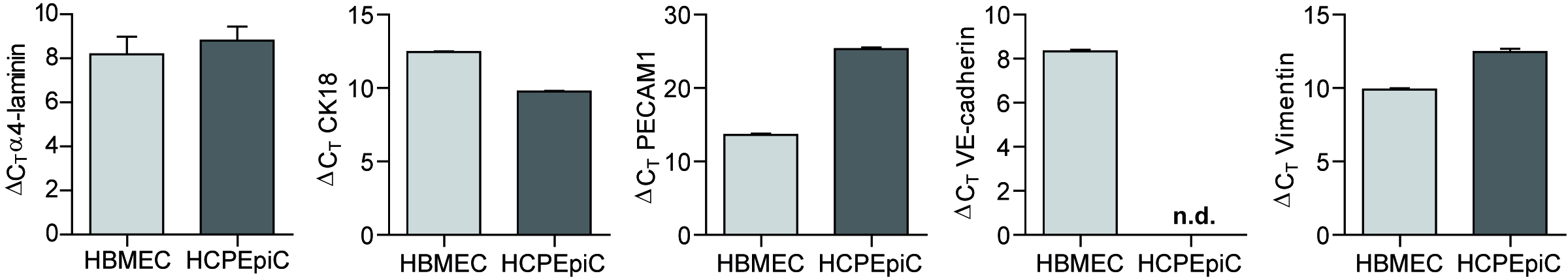

Supplement: Supplementary file 3 — Figure S3. Characterization of human brain-derived microvascular endothelial cells and fibroblasts derived from human choroid plexus cells. mRNA levels of laminin α4, cytokeratin 18 (CK18), PECAM1, VE-cadherin, and vimentin in primary human brain-derived microvascular endothelial cells (HBMEC) and fibroblasts originated from primary human choroid plexus epithelial cells (labeled as HCPEpiC) were quantified by real-time PCR, revealing the lack of epithelial markers by HCPEpiC and confirming their fibroblastic nature. n.d. = not detected (TIF 4578 kb) [file 12974_2018_1276_MOESM3_ESM.tif]

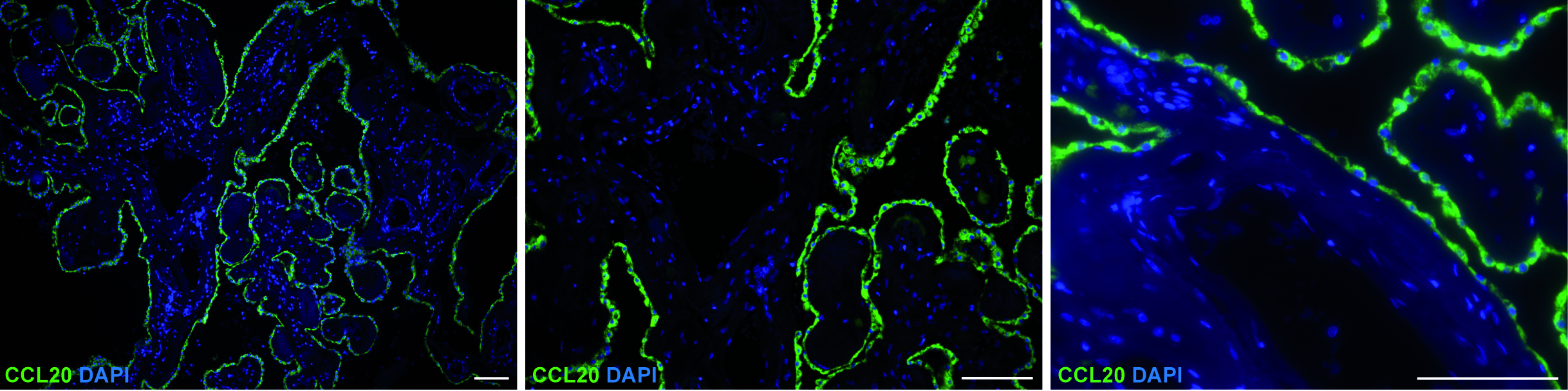

Supplement: Supplementary file 4 — Figure S4. CCL20 localization on human choroid plexus tissue. CCL20 staining (green) on human choroid plexus epithelium in control CNS tissue samples. Nuclear staining (DAPI) is shown in blue. Scale bars = 100 μm. (TIF 6336 kb) [file 12974_2018_1276_MOESM4_ESM.tif]
